# Diagnostic accuracy of the 5.07 monofilament test for diabetes polyneuropathy: influence of age, sex, neuropathic pain and neuropathy severity

**DOI:** 10.1136/bmjdrc-2023-003545

**Published:** 2023-11-20

**Authors:** Øystein Dunker, Martin Uglem, Marie Bu Kvaløy, Sissel Løseth, Ina Elen Hjelland, Sara Maria Allen, Maria Dehli Vigeland, Inge Petter Kleggetveit, Trond Sand, Kristian Bernhard Nilsen

**Affiliations:** 1 Department of Research and Innovation, Oslo University Hospital, Oslo, Norway; 2 Faculty of Medicine, Institute of Clinical Medicine, University of Oslo, Oslo, Norway; 3 Department of Neurology and Clinical Neurophysiology, Oslo University Hospital, Oslo, Norway; 4 Department of Neuromedicine and Movement Science, Norwegian University of Science and Technology Faculty of Medicine and Health Sciences, Trondheim, Norway; 5 Department of Neurology and Clinical Neurophysiology, St Olavs Hospital, Trondheim University Hospital, Trondheim, Norway; 6 Department of Neurology, Section of Clinical Neurophysiology, Stavanger University Hospital, Stavanger, Norway; 7 Department of Neurology and Clinical Neurophysiology, University Hospital of North Norway, Tromsø, Norway; 8 Department of Clinical Medicine, The Artic University of Norway, Tromsø, Norway; 9 Department of Clinical Neurophysiology, Haukeland University Hospital, Bergen, Norway

**Keywords:** diabetic neuropathies, diagnostic techniques and procedures

## Abstract

**Introduction:**

There is a need for simple and cheap diagnostic tools for diabetic polyneuropathy (DPN). We aimed to assess the diagnostic accuracy of the 5.07/10 g monofilament test in patients referred to polyneuropathy assessments, as well as to examine how disease severity, age, sex and neuropathic pain (NP) impact diagnostic accuracy.

**Research design and methods:**

Five Norwegian university hospitals recruited patients with diabetes aged 18–70 referred to neurological outpatient clinics for polyneuropathy assessments. The 5.07/10 g Semmes-Weinstein monofilament examination (SWME) was validated against the Toronto consensus for diagnosing diabetic neuropathies; the results were stratified by age, sex and NP. Disease severity was graded by a combined nerve conduction study (NCS) Z-score, and logistic regression was applied to assess whether disease severity was a predictor of diagnostic accuracy.

**Results:**

In total, 506 patients were included in the study. Global sensitivity was 0.60 (95% CI 0.55, 0.66), specificity 0.82 (95% CI 0.75, 0.87), positive and negative predictive values were 0.86 (95% CI 0.81, 0.90) and 0.52 (95% CI 0.46, 0.58), respectively, positive and negative likelihood ratios were 3.28 (95% CI 2.37, 4.53) and 0.49 (95% CI 0.42, 0.57), respectively. The SWME was less sensitive in females (0.43), had lower specificity in patients with NP (0.56), and performed worse in patients ≥50 years. NCS-based disease severity did not affect diagnostic accuracy (OR 1.15, 95% CI 0.95, 1.40).

**Conclusions:**

This multicenter study demonstrates poor diagnostic performance for the 5.07/10 g SWME in patients with diabetes referred to polyneuropathy assessments; it is particularly unsuited for female patients and those with NP. The diagnostic accuracy of the SWME was not influenced by NCS-based disease severity, demonstrating that it does not perform better in patients with later stages of DPN. We do not recommend the use of the 5.07/10 g monofilament in the evaluation of patients with diabetes referred to polyneuropathy assessments.

**WHAT IS ALREADY KNOWN ON THIS TOPIC**

- The monofilament test is widely used, but its diagnostic accuracy for diabetic polyneuropathy (DPN) is still unclear.

**WHAT THIS STUDY ADDS**

- We show that the monofilament test is particularly unsuitable for female patients and patients with neuropathic pain, and that disease severity does not impact the test’s diagnostic accuracy.

**HOW THIS STUDY MIGHT AFFECT RESEARCH, PRACTICE OR POLICY**

- The monofilament test should not be used to diagnose DPN, nor be used as an inclusion tool in diabetes research.

## Introduction

Diabetes is on the rise, now estimated to affect over 10% of the world’s adult population,^1^ while another 7.5% are at high risk of developing diabetes due to impaired glucose intolerance or metabolic syndrome.^2^ Yearly global healthcare expenditures for adult patients with diabetes are nearing US$1 trillion, or about one-tenth of the world’s total health spending.^1^ Diabetic polyneuropathy (DPN) is the most common complication of diabetes; it affects as many as half of patients^3^ and accounts for a considerable part of diabetes morbidity, mortality and financial burden.^4–6^

There is currently no treatment for DPN. As such, the goal of early diagnosis is to delay progression of the disease and prevent related complications (eg, foot ulcers, falls or amputations), as well as help patients manage neuropathic pain and other symptoms.^5 7–9^ The most widely accepted reference standard for diagnosing DPN is nerve conduction studies (NCS) in combination with a clinical examination.^10–12^ However, NCS is resource intensive,^10 13^ which makes it poorly suited for both small-scale and large-scale use (eg, primary care physicians or large-scale screening of diabetes populations). Thus, there is a need for simple, but sufficiently accurate diagnostic tools for DPN.

A 5.07/10 g Semmes-Weinstein monofilament examination (SWME) is commonly recommended for patients at risk for DPN.^8 14 15^ SWME is inexpensive and easy to use and is widely applied for both screening and diagnostic purposes. Nevertheless, due to large variation in test procedures and interpretations in the literature, it is still unclear whether the diagnostic accuracy of SWME is sufficient to warrant its place in the health professional’s toolkit.^16^

The mechanical detection threshold under the feet decreases with age,^17^ and may also be influenced by sex^18^ and neuropathic pain,^19^ threatening the validity of a one-size-fits-all monofilament. Furthermore, it has been hypothesized that SWME predominantly detects late-stage polyneuropathy.^10 11 20^ If true, the SWME would neither contribute to early diagnosis nor facilitate early interventions, thus reducing its clinical relevance.

Therefore, the general aim of this study was to validate the Norwegian SWME protocol^15^ in patients with diabetes referred to polyneuropathy assessments. Specific objectives were to provide estimates for diagnostic accuracy, to examine whether DPN severity is a predictor of SWME performance, and to quantify the difference in diagnostic accuracy between sexes, age strata and for patients with neuropathic pain. This study is part of a large multicenter study including five clinical neurophysiology departments at Norwegian university hospitals.

## Methods

### Overview, approval and consent

We employed a cross-sectional design to ascertain the diagnostic accuracy of the SWME in patients with diabetes, referred to polyneuropathy assessments at five different neurological outpatient clinics in Norway. The widely used Toronto consensus on diagnostic criteria for DPN served as the reference standard.^12^ We graded the severity of DPN using a combined NCS score. The study follows the Standards for Reporting Diagnostic accuracy studies (STARD) guidelines.^21 22^ It is a prospective study: data collection was planned before index tests and reference tests were performed. All subjects gave informed consent prior to inclusion.

### Study sample and recruitment procedure

The sample is part of a larger Norwegian multicenter study where patients aged 18–70 referred to neurological hospital outpatient clinics for polyneuropathy assessment were asked to participate, that is, a convenience sample. When a patient was referred to the hospital by a diabetes clinic or their primary care provider, they would receive an invitation letter, questionnaire and consent form by mail. The initial exclusion criteria were acute polyneuropathies (eg, acute inflammatory demyelinating polyradiculopathy, acute motor axonal neuropathy), nerve entrapment without polyneuropathy, limited capacity to give informed consent (eg, language barrier, dementia, psychiatric illness) and patients being too sick to participate (eg, bed ridden, high fever), of which the distribution can be found in a previously published study on the same material.^23^ The present study only analyzed the patients with diabetes mellitus. To reflect clinical practice, patients with likely predominant small-fiber polyneuropathy were not excluded from the main analysis.

Five hospitals participated in the data collection between May 2017 and December 2022: Oslo University Hospital; Haukeland University Hospital, Bergen; Stavanger University Hospital; St Olavs Hospital, Trondheim University Hospital, Trondheim; and University Hospital of North Norway, Tromsø.

### The 5.07/10 g SWME

The SWME was performed using a 5.07 monofilament (Aesthesio, DanMic Global, California, USA) that bows at roughly ‘10g [axial] force’ when applied at a right angle (gram-force is a deprecated, non-standard unit, equaling ~0.1 N). More accurately, it is the local *stress* or pressure that leads to the nerve response, thus the 5.07 monofilament tests cutaneous mechanical sensation at ~0.066–0.095 N/mm^2^ (in this case Ø0.475 mm=0.17 mm^2^),^24^ or ~66–95 kPa. The monofilament was replaced if it had a noticeable bend or was otherwise damaged.

The SWME followed the Norwegian guidelines by the Norwegian Directorate of Health.^15^ The patient was supine on an examination table for the examination. First, the physician demonstrated the feel of the monofilament by applying it to the patient’s hand. Then, the patient was instructed to close their eyes, and the physician applied the monofilament again and this time asked whether the patient could feel the monofilament touching the skin. Four sites were subsequently tested on the plantar aspect of each foot, with care to avoid calluses: the heads of the first, third and fifth metatarsals, and on the distal phalanx of the first toe. The sites were tested in random order with variable rhythm. If the patient did not say ‘yes’ at a site, the physician came back to this site once more.

### Reference standard

The reference standard was the Toronto consensus on diagnosing diabetic neuropathies.^12^ The definitions of minimal criteria for DPN are: *subclinical DPN* (no signs or symptoms, but abnormal NCS or validated small-fiber test); *possible DPN*, requiring either symptoms (negative or positive symptoms; eg, numbness, pain or paresthesia) or signs (symmetrical decreased sensation or decreased/absent ankle reflexes); *probable DPN*, which requires a combination of symptoms and signs (two or more of neuropathic symptoms, decreased distal sensation, and decreased/absent ankle reflexes); and *confirmed DPN,* which necessitates the presence of an abnormality of NCS or a validated test for small-fiber neuropathy, as well as either symptoms or signs of polyneuropathy. In accordance with the Norwegian national guidelines for clinical neurophysiology,^25^ at least *two* nerves of different roots had to be abnormal to constitute a positive NCS finding (three if abnormal medial plantar nerve).

To avoid incorporation bias, the results of the SWME were not entered into the reference standard; instead, the clinicians used a brush, pin and/or cotton swab to test for the item ‘decreased sensation’. In line with the Toronto consensus, and to reduce the number of false positives in the reference standard, only patients with *confirmed DPN* were regarded as true positives. Since confirmatory tests for DPN are not always available in real-life clinical practice, probable DPN was included as true positives in a subanalysis.

### Assessment procedure

History taking, clinical examination and NCS were performed as part of routine assessments for polyneuropathy. The NCS were performed in concordance with the Norwegian national guidelines.^25^ A minimum of two sensory nerves in the feet were tested (sural nerve and medial plantar nerve), as well as two motor nerves (tibial nerve and peroneal nerve), including F-responses. If the NCS findings were not clear, one extra sensory nerve was tested (superficial peroneal nerve).

For small-fiber neuropathy, the Toronto consensus requires normal (sural) NCS and either altered intraepidermal nerve fiber density or abnormal thermal detection thresholds in the feet. Quantitative thermal testing of the lower extremities was used as the primary confirmatory test for small-fiber lesions; skin biopsy was used in the opaquest cases at Oslo University Hospital, and accounts for only 5 of 45 small-fiber diagnoses. For quantitative thermal testing, only detection thresholds were used to assess abnormality.^26^ We employed the method of limits: the baseline temperature was 32°C, ramp rate 1°C/s and thermode size 9–12 cm^2^, as per the national guidelines^25^ and the hospitals’ own protocol.

Local reference values for both NCS and quantitative thermal testing were applied when possible. Alternatively, reference values from Powerpack (Stefan Stålberg Software, Helsingborg, Sweden),^27^ Hafner *et al*^28^ or the national guidelines (quantitative thermal testing) were used.

History taking and clinical examination, including the SWME, were performed by a consultant clinical neurophysiologist. The NCS were performed on the same day as the clinical examination, most often by a technician, but were in some instances done by the consultant physician. Since the tests were conducted as part of a normal polyneuropathy assessment, the physicians were not systematically blinded from the NCS results, although at some hospitals the SWME was conducted by the physician either before NCS or without prior knowledge of the NCS results.

### Grading of the severity of DPN

As a measure of DPN severity, we converted NCS results into Z-scores, including the tibial and peroneal motor nerves (distal amplitude, conduction velocity, F-M min latency), the sural nerve (amplitude and conduction velocity), the peroneal superficial nerve (amplitude and conduction velocity), and the medial tibial plantar nerve (amplitude), in line with recent work on the subject.^29^ We then averaged the Z-scores into a single, continuous score, a *compound Z-score*. A total of 247 patients were included in this subanalysis (aged 54.8±10.7 years, 63% male) from Oslo University Hospital (n=110), Stavanger University Hospital (n=60) and Trondheim University Hospital (n=77). The reference material and Z-score calculation is described in detail in online supplemental file 1.

10.1136/bmjdrc-2023-003545.supp1 Supplementary data

### Neuropathic pain

Patients were assessed for neuropathic pain *in the feet* by use of the The International Association for the Study of Pain's Neuropathic Pain Special Interest Group's (NeuPSIG) criteria, described in detail elsewhere.^30^ The NeuPSIG criteria classify patients by the level of confidence that neuropathic pain is present, graded as *unlikely, possible, probable* and *definite* neuropathic pain. Patients were dichotomized as having neuropathic pain (*probable, definite*) or not (*unlikely, possible*). We included *probable* cases in the neuropathic pain group since *definite* requires confirmatory tests that are not always readily available in the clinical setting.

### Sample size

Sample size calculations for the diagnostic accuracy of the SWME were not performed, as the data for the present study were already collected as part of a larger multicenter study. However, sample size calculation for the logistic regression was conducted to ascertain how much raw NCS data on included patients needed to be retrieved from hospital databases. This calculation was based on the rule of thumb of Peduzzi *et al*,^31^ that is, *n*=10*number of covariates/*smallest proportion of positive or negative cases in the population studied*. Clinical experience suggested roughly 65% positive cases. The predictor of interest was a combined NCS Z-score but adjusted for age and sex. We therefore approximated *n*=10*3/0.35=86 patients. When this equation provides a ‘low’ number, the sample size should conservatively default to 100.^32^ Thus, we required minimum 100 patients for the logistic regression.

### Missingness, imputation and quality control of raw NCS data

In total, 11.9% of NCS data points were missing from 109 of 247 patients with incomplete studies. Of the 11 measures included, eight had missing data (tibial, peroneal and sural amplitudes were complete). Of note, peroneus superficial conduction velocity had a very high amount of missing (43.8%), partly due to non-registerable sensory nerve action potentials (20.5%), but also as a result of its infrequent use at one of the hospitals (Stavanger). Further, the total missing of sural conduction velocity and peroneus F-waves was 16.1% and 11.6%, respectively, while the remainder were missing ≤10% of data points. The raw NCS data were cleaned and imputed; the process is described in detail in online supplemental file 1.

### Analyses of diagnostic accuracy

For the main analysis, the number of sensate sites during the examination was summed up to a total ranging from 0 (worst) to 8 (best). The patients were then dichotomized by number of sensate sites: 0–6 meant a positive test for DPN, while 7–8 meant a negative test.^15^ If the SWME or reference test could not be performed as per the protocol, the patient was not included in the analysis. We assessed the diagnostic accuracy of the SWME by calculating sensitivity, specificity, positive/negative predictive values (PPV/NPV), positive/negative likelihood ratios (LR+/LR−) and proportion of correctly classified patients. The overall discriminative ability of the SWME was determined by analysis of the (area under the) receiver operating characteristic curve (ROC AUC).

The clinical value of the SWME was assessed mainly through predictive values and likelihood ratios. Predictive values tell the clinician how likely it is that a given test result is true, while likelihood ratios convey how the SWME affects post-test probability of disease. For likelihood ratios, the effect of the test on post-test probability was interpreted as s*mall, rarely important* (1–2 and 0.5–1), *small, but sometimes important* (2–5 and 0.5–0.2), *moderate* (5–10 and 0.1–0.2) and *large, often conclusive* (>10 and <0.01).^33^ The ROC AUC value was considered to be *non-discriminative* (0.5–0.6), *poor* (0.6–0.7), *acceptable* (0.7–0.8), *excellent* (0.8–0.9) or *outstanding* (>0.9).^34^

### The effect of age, sex, neuropathic pain and DPN severity on the SWME

Contingency tables were also made for subgroups: diagnostic accuracy was calculated for males, females, patients with neuropathic pain, and the age groups <50 and ≥50 years old. An ROC curve was generated for each subgroup, and Youden’s Index (maximum combined sensitivity+specificity−1) was marked. Logistic regression was performed to assess whether the NCS-based severity of DPN could predict the outcome of the monofilament test. The dependent variable was correct classification by the monofilament test, while the independent variable was our NCS measure of DPN severity, that is, the compound Z-score of 11 NCS measures from the lower extremities. The model was adjusted for age, sex and neuropathic pain. The Hosmer-Lemeshow test for goodness of fit was performed for each model. A p value <0.05 was considered significant.

## Results

In total, 506 patients were included in the study, of which 66% had confirmed DPN by the reference standard and 54% had neuropathic pain. There were no adverse events from the examinations. Demographics and clinical variables are presented in table 1.

**Table 1:** Patient demographics and clinical variables (n=506)

	All (n=506)	Males (n=307)	Females (n=199)	<50 years (n=139)	≥50 years (n=367)	Neuropathic pain (n=273)
Age, years, mean (SD)	55 (11)	57 (10)	53 (12)	41 (7)	61 (6)	56 (10)
Sex, female, n (%)	199 (39)	–	–	68 (49)	129 (35)	107 (38)
Diabetes type, n (%)						
Type 1	118 (23)	62 (20)	56 (28)	70 (50)	48 (13)	45 (16)
Type 2	321 (63)	199 (65)	122 (61)	56 (40)	265 (72)	193 (71)
Do not know	67 (13)	46 (15)	21 (11)	13 (9)	54 (15)	11 (4)
HbA1c, mmol/mol, mean (SD)	49 (50)	52 (19)	48 (22)	48 (24)	51 (18)	52 (20)
Toronto DPN consensus classification, n (%)^12^						
Normal	43 (8)	12 (4)	31 (15)	17 (12)	22 (6)	0 (0)
Possible	67 (13)	36 (12)	31 (15)	25 (18)	42 (11)	0 (0)
Probable	64 (13)	31 (10)	33 (17)	21 (15)	47 (13)	39 (14)
Confirmed	332 (66)	228 (74)	104 (53)	76 (55)	256 (70)	234 (86)
Toronto Clinical Neuropathy Score (TCNS), mean (SD)^54^	10 (5)	10 (5)	8 (5)	8 (5)	10 (5)	12 (4)
Small-fiber neuropathy, n (%)	45 (9)	21 (7)	23 (12)	11 (8)	33 (9)	39 (14)
Neuropathic pain*, n (%)^30^	277 (55)	169 (55)	106 (53)	69 (50)	208 (57)	–

*Probable or definite neuropathic pain by the The International Association for the Study of Pain's Neuropathic Pain Special Interest Group's (NeuPSIG) criteria.

DPN, diabetic polyneuropathy .

### Diagnostic accuracy of SWME for DPN

**Figure 1:**
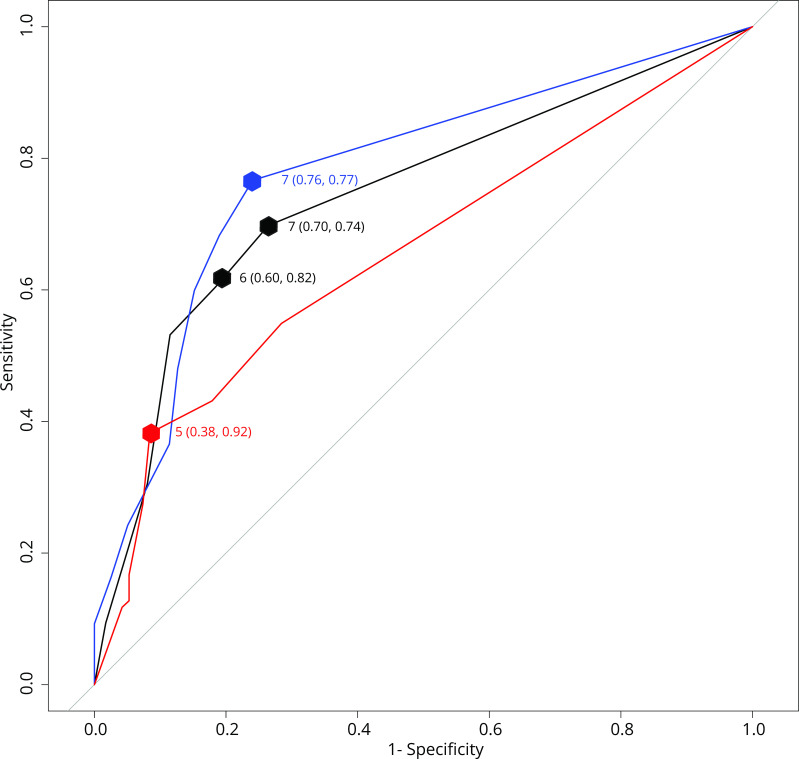
Area under the receiver operating characteristic curve (AUC) for the Semmes-Weinstein monofilament examination (SWME) with markers for some selected cut-offs (with their associated sensitivity and specificity in parentheses). The curve for the males is drawn in blue, females in red and the combined group in black. The preselected primary cut-off (six or less sensate sites) is marked for the combined group, as well as Youden’s Index for all groups (maximum combined sensitivity+specificity−1).

The SWME had poor sensitivity and moderate specificity for diabetes polyneuropathy (table 2). With high prevalence of confirmed DPN, the PPV was adequate, while the NPV was of little use. The modest likelihood ratios mean that the test only had a small, rarely important effect on post-test probability. The overall discriminate ability of SWME was acceptable, with a total ROC AUC of 0.74 (95% CI 0.70, 0.78) (figure 1).

**Table 2:** Diagnostic accuracy of the 5.07/10 g monofilament test

		Toronto consensus				Point estimate (95% CI)
		Positive	Negative	Total		
**Monofilament**	Positive	200	32	232	Sensitivity	0.60 (0.55, 0.66)
	Negative	132	142	274	Specificity	0.82 (0.75, 0.87)
	Total	332	174	506	PPV	0.86 (0.81, 0.90)
					NPV	0.52 (0.46, 0.58)
					LR+	3.28 (2.37, 4.53)
					LR−	0.49 (0.42, 0.57)
					Area under the ROC curve	0.74 (0.70, 0.78)
					Correctly classified proportion	0.68 (0.63, 0.72)

LR−, negative likelihood ratio; LR+, positive likelihood ratio; NPV, negative predictive value; PPV, positive predictive value; ROC, receiver operating characteristic.

The diagnostic accuracy changed when stratified for sex (table 3). On average, females were younger than the males (53±12 years old vs 57±10 years old, p<0.01). When only females were included, SWME sensitivity was reduced by 17 percentage points, while the specificity was largely unchanged. This entails that while the PPV was still at 75% in females due to the high specificity and disease prevalence, the test now had a false negative rate of ~60%, reducing the number of true positives dramatically.

The monofilament test performed relatively similarly in age groups above (inclusive) and below 50 years. Specificity, NPV and LR+ were higher in the younger group, the latter of which approached a moderate effect on post-test probability. In patients with neuropathic pain, we found lower specificity (0.56) and subsequently lower NPV (0.20) due to an increased proportion of false positives.

For patients with predominant small-fiber neuropathy (n=45), 34% were insensate to the SWME. Excluding these patients did not result in clinically meaningful changes to diagnostic accuracy (sensitivity 0.65, specificity 0.83, PPV 0.87, NPV 0.58).

Including *probable DPN* cases as true positives had a small impact on diagnostic accuracy (sensitivity 0.56, specificity 0.91, PPV 0.96, NPV 0.36, LR+ 6.17, LR− 0.48).

**Table 3:** Diagnostic accuracy of the SWME by age, sex and neuropathic pain

	Point estimates (95% CI)					
	All (n=506)	Male (n=307)	Female (n=199)	<50 years (n=134)	≥50 years (n=372)	Neuropathic pain (n=273)
Sensitivity	0.60 (0.55, 0.66)	0.68 (0.62, 0.74)	0.43 (0.33, 0.53)	0.54 (0.41, 0.65)	0.62 (0.56, 0.68)	0.62 (0.55, 0.68)
Specificity	0.82 (0.75, 0.87)	0.81 (0.71, 0.89)	0.83 (0.73, 0.90)	0.89 (0.78, 0.95)	0.78 (0.69, 0.85)	0.56 (0.40, 0.72)
PPV	0.86 (0.81, 0.90)	0.91 (0.86, 0.95)	0.75 (0.62, 0.86)	0.84 (0.71, 0.94)	0.87 (0.81, 0.91)	0.90 (0.84, 0.94)
NPV	0.52 (0.46, 0.58)	0.47 (0.38, 0.56)	0.54 (0.45, 0.63)	0.63 (0.52, 0.73)	0.47 (0.40, 0.55)	0.20 (0.13, 0.28)
LR+	3.28 (2.37, 4.53)	3.60 (2.26, 5.72)	2.52 (1.49, 4.27)	4.82 (2.32, 10.01)	2.84 (1.97, 4.09)	1.42 (0.98, 2.06)
LR−	0.49 (0.42, 0.57)	0.39 (0.31, 0.49)	0.69 (0.56, 0.84)	0.52 (0.40, 0.68)	0.48 (0.40, 0.58)	0.67 (0.49, 0.93)
Area under the ROC curve	0.74 (0.70, 0.78)	0.78 (0.72, 0.83)	0.66 (0.59, 0.72)	0.72 (0.65, 0.79	0.74 (0.70, 0.80)	0.72 (0.65, 0.79)
Correctly classified proportion	0.68 (0.63, 0.72)	0.72 (0.66, 0.77)	0.61 (0.53, 0.68)	0.70 (0.62, 0.78)	0.67 (0.62, 0.72)	0.61 (0.55, 0.67)

LR−, negative likelihood ratio; LR+, positive likelihood ratio; NPV, negative predictive value; PPV, positive predictive value; ROC, receiver operating characteristic; SWME, Semmes-Weinstein monofilament examination.

### Polyneuropathy severity as predictor of SWME test result

Of the 247 patients with raw NCS data, 156 (63%) had polyneuropathy by the reference standard. The median compound Z-score for the NCS measures was 0.89 (range −0.76, 6.69; IQR 2.04). NCS-based disease severity (compound NCS Z-score) was not a significant predictor of how well the SWME performed (table 4). The Hosmer-Lemeshow test for goodness of fit was non-significant for both models.

**Table 4:** Multivariate logistic regression: association between NCS-based disease severity and the diagnostic accuracy of the SWME

Variable	Model 1			Model 2		
	OR	95% CI	P value	OR	95% CI	P value
Compound NCS Z-score	1.20	0.99, 1.45	0.06	1.20	0.95, 1.54	0.14
Age (years)				1.01	0.98, 1.05	0.34
Sex (female)				0.80	0.41, 1.54	0.50
Neuropathic pain				0.55	0.27, 1.06	0.08

NCS, nerve conduction study; SWME, Semmes-Weinstein monofilament examination.

## Discussion

In this large, multicenter study on SWME in patients with diabetes referred to a polyneuropathy assessment, we found acceptable discriminative ability, PPV and specificity, but poor sensitivity, NPV and overall effect on post-test probability of disease. We showed that the 5.07 monofilament test is particularly unsuitable for female patients and patients with neuropathic pain, and that NCS-based DPN severity did not impact test performance.

The main results are in concordance with previous findings, which tend toward low sensitivity and moderate to high specificity for the SWME.^16^ As a simple test, the 5.07 monofilament is relatively specific, but high specificity is of little use by itself. Due to low sensitivity, almost half of patients with DPN are overlooked, diminishing the clinical value of a negative result. A possible explanation for the poor sensitivity could be our use of a sensitive reference standard. It is unlikely that a simple test for foot sensibility is as sensitive for DPN as more detailed clinical examinations, with or without NCS. Still, even simple tests should be validated against rigorous reference standards to gauge their true accuracy.

For the clinician, the test’s predictive values are more important when assessing a patient than the test’s credentials against a reference standard. Although there is currently no approved treatment for DPN, regular foot examinations, exercise, improved glycemic control and proper footwear are recommended management strategies.^7 35^ With early diagnosis and proper care, one can likely reduce the risk of serious sequelae from, for example, ulcers or falls. It is also important to note that although the SWME underperforms in assessing DPN, it may still be useful in predicting foot ulcers.^36^ In addition, receiving a true DPN diagnosis empowers the patient to take an active part in the disease management. In this scenario, a moderate PPV and high NPV would be ideal.^37^ A few more false positives would make a good trade against a reduction in false negatives, the latter of which entails that preventive action is delayed and the disease may progress further than it otherwise would have. In this light, the SWME adds little value for the clinician, since interpreting a negative result can be likened to pure chance. Therefore, we echo previous sentiments that the sole use of SWME is not sufficient for DPN assessment,^16 35 38^ neither for clinical nor research purposes. We would also question whether the diagnostic accuracy of the 5.07 monofilament supports its inclusion in larger diagnostic batteries.

The 5.07 monofilament did not perform well in females. In fact, our main result of 60% sensitivity is likely somewhat inflated due to the fact that our sample contains 50% more males than females. When stratified by sex, the proportion of correctly classified female patients is significantly reduced, with a false negative rate of well more than half. Few have investigated the effect of sex on SWME: Pambianco *et al*^39^ did not find any difference between sexes with the 5.07 monofilament. This contrasting finding could perhaps be attributed to differences in protocol, as they only tested the dorsum of the big toe. Two studies (on the same dataset) from the German research network on neuropathic pain did not identify a significant difference on the group level between sexes for mechanical detection threshold in the feet with a set of modified monofilaments.^17 18^ However, they did find higher variability and an age effect in males; since valid reference limits must account for variability, their findings suggest that different monofilaments should be used for males and females. Although there was an age difference between sexes in our study, it is unlikely to be large enough to have meaningfully impacted the results.^18^ This is supported by our subgroup analysis which showed little difference in overall discriminative ability between those above and below 50 years of age. If there is a true difference in mechanical detection sensitivity between the sexes, a possible solution may be to use a monofilament that exerts less pressure in female patients. Indeed, monofilaments labeled as 4.31 (~2 g) or 4.17 (~1 g) have been proposed as more sensitive tests,^40–42^ and are in fact much closer to the expected detection threshold in healthy adults.^43–45^ Comparatively, the 5.07 monofilament represents a sensory threshold tens of times greater than the expected mean in healthy individuals.^24 44^ As a result, the 5.07 monofilament lends itself to being more sensitive in patients with poorer cutaneous sensation, either as a result of advanced neuropathy or, possibly, being male. For the SWME to be worthwhile, it needs to be simple, accessible and quick to administer, which precludes the use of the 20-set monofilament kit to determine exact thresholds. Future studies may seek to properly assess the diagnostic properties of monofilaments that buckle under less force, since they are likely to increase the sensitivity in female patients.

While the most recent review of the literature did not recommend the 5.07 SWME for screening purposes,^16^ there has been an expectation that it would perform better in patients with more pronounced thick-fiber lesions, that is, in later stages of DPN. With the natural progression of DPN in the feet, it follows that the diagnostic accuracy of the SWME should improve in the later stages of disease.^10 20^ We found no such association between NCS-based DPN severity and the diagnostic accuracy of the SWME. Since our sample was drawn from recently referred patients, we cannot rule out that a sample with a higher incidence of severe nerve lesions could yield a different result. However, the effect is unlikely to be marked—our dataset is well populated over quite a range of DPN severity. In addition, the monofilament test is most appropriately applied early, for example, for screening or in early diagnostic settings, when patients have similar or even less severe nerve lesions than in the present study, thus rendering the point somewhat moot.

Lastly, the SWME performed worse in patients with neuropathic pain. Although relatively few in absolute numbers, the proportion of false positives was higher than in the main analysis. This means that patients with neuropathic pain may be less sensitive to static cutaneous pressure from a monofilament at the site of pain, yet this is not directly reflected in the subanalysis’ sensitivity score. One possible explanation could be that the test is affected by the presence of dysesthesia, which is common in patients with neuropathic pain.^46^ Another explanation is that the findings are exaggerated, and more a result of how we dichotomize DPN (the ‘false positives’ have ‘probable DPN’). Since many patients seek help when symptoms emerge, the clinician should be aware that symptoms of neuropathic pain may impact the SWME and interpret the results accordingly until more is known on the subject.

The present study is well powered with several strengths. It is a large multicenter study, including five Norwegian university hospitals. We employed a rigorous reference standard for diagnosing DPN, which should reduce the number of false classifications greatly. Our use of a compound Z-score for assessing DPN severity gives new insight into how the diagnostic accuracy of SWME is (un)affected by changes in nerve function.

Some limitations should also be mentioned. Our measure of DPN disease severity was based solely on NCS. This does not necessarily reflect clinical severity, which in our study was fairly low (cf Toronto Clinical Neuropathy Score). However, a compound NCS Z-score measures overall nerve function and is a good indicator of DPN progression in the myelinated nerves that respond to monofilament pressure. We did not systematically blind the consultant neurophysiologist for the NCS results, as described in detail in the Methods section. However, if some NCS results were known before the SWME, this could only have improved our results, as NCS is the far better test for large-fiber lesions. Thus, our conclusion of poor diagnostic accuracy would not change, but would be, if anything, slightly understated. Furthermore, the Norwegian SWME protocol should perhaps have been validated as a screening tool in primary care, and not as a diagnostic tool in tertiary care. Still, if the 5.07 monofilament test cannot distinguish between patients with and without DPN in a high-prevalence setting, in the hands of a trained neurophysiologist, it is unlikely to perform better in primary care. In addition, the Norwegian SWME protocol involves testing mechanical detection thresholds at four sites on the plantar aspect of each foot. There is currently no agreed upon standard for how many sites to test, but testing a different number of sites is unlikely to meaningfully change the diagnostic accuracy.^16 47^ Lastly, we cannot be certain that the monofilaments used in this study are in fact ‘10g’, as we did not validate them on-site and throughout the testing period. On one hand, it is a known issue that the buckling force of the monofilaments may vary quite a bit from the manufacturer, and may also change over time, for example, as a response to repeated use and changes in temperature and humidity.^48–51^ On the other hand, assurances of correct buckling force would not meaningfully affect the external validity of the study, as buckling force variability is likely to be an issue everywhere.

In conclusion, the SWME with a 5.07 monofilament showed acceptable overall discriminative ability by ROC AUC, with low sensitivity and acceptable specificity in patients referred to a polyneuropathy assessment, when compared with the Toronto consensus for diagnosing DPN. The clinical value of the SWME for this population is limited: the high rate of false negatives with almost half of DPN cases missed, and the subsequent low NPV renders negative results meaningless. Overall diagnostic accuracy was similar when stratifying by age, but the SWME performed worse in patients with neuropathic pain, and in females. Future studies on SWME should properly assess whether monofilaments that exert less pressure may be more appropriate for female patients; a change in course could also be warranted as modern point-of-care devices become more affordable.^52 53^ The SWME did not perform better in patients with more pronounced thick-fiber nerve lesions. We do not recommend that the 5.07/10 g monofilament is used as a stand-alone test, nor as part of a test battery in the evaluation of patients with diabetes referred to a polyneuropathy assessment.

## Data Availability

Data are available upon reasonable request. Qualified researchers may request access to an anonymized version of the original dataset, as well as the analysis-ready dataset for logistic regression, for up to 2 years after publication.
