# Supplementary material for: Diagnostic accuracy of the 5.07 monofilament test for diabetes polyneuropathy: influence of age, sex, neuropathic pain and neuropathy severity
Source: BMJ Open Diabetes Res Care. 2023 Nov 20;11(6):e003545. doi: 10.1136/bmjdrc-2023-003545 (PMC10660161; doi:10.1136/bmjdrc-2023-003545)
Supplement: Supplementary data [file bmjdrc-2023-003545supp001.pdf]

Supplemental files 1

Data-cleaning and imputation

First, two researchers (ØD and KBN) went through all the raw NCS data to identify [technical] errors in the material or measures that could not necessarily be trusted, e.g. F-waves below 30m/s in the lower limbs, motor amplitudes below 0.5 mV or registered F-waves/conduction velocities dependent on the latter. Such values had their markers in the EMG software double-checked and re-done if necessary, and patient journals were examined when required. Erroneous entries were removed and marked for imputation by the most appropriate imputation step of the two described below.

Second was the imputation process. Regular (multiple) imputation by linear regression or predictive mean matching was deemed invalid: although there was no overall discernable pattern of missingness, much of the missing data was likely pathological and therefore created a type of censored dataset (e.g. missing conduction velocity or F-waves for a nerve with non-registerable amplitudes). This meant that linear regression would underestimate the missing values and impute within a very narrow range at the model's tail, and predictive mean matching would lack valid donors. Most importantly, we had information regarding how the missing data was likely to look, which needed to be part of the imputation model for the results to make sense. Therefore, we imputed the missing data in three steps:

1. Non-registerable amplitudes were imputed with random, normally distributed values between 0 and 0.1, to avoid stacking of maximum Z-scores.
2. Missing conduction velocities and F-waves due to non-registerable amplitudes (the vast majority of missing data) were imputed as follows: For each patient, the limit of abnormality (defined as ±2SD) was calculated based on our reference material. For conduction velocities, the imputed Z-score was constrained between Z = 2 (positive sign is abnormal) and the Z-score of a conduction velocity 30% below the expected value (*ZLimit*)[1]. For F-waves the constraints were between Z = 2 and an increase in latency of 20%[1]. The final Z-score imputed was randomly calculated within these parameters, and then multiplied with the normal distribution, to better reflect the expected values in a large sample. For example

$$\text{Imputed } Z_{CV} = 2 + (\text{Zlimit} - 2) * (1 - \text{Random normally distributed value between 0 and 1})$$

Data still missing (<1%), i.e., due to technical errors or other random effects were then imputed by fully conditional specification (chained equations), with 10 iterations, based on predictive mean matching with a donor pool of five (SPSS v25). Twenty imputations were run and the average value was imputed.

Z-score calculations and grading of DPN severity

As a measure of DPN severity, we converted NCS results into Z-scores, and then averaged the Z-scores into a single, continuous score, a *compound Z-score*. A total of 247 patients were included in this sub-analysis (aged 54.8 ± 10.7 years, 63% male) from Oslo (n = 110), Stavanger (n = 60) and Trondheim (n = 77) university hospitals. The Z-scores were calculated as *Z =* (*Observed NCS measure – predicted NCS measure*) / *root mean square error of the linear regression model from the reference material*. Z-score signs were adjusted so that abnormal results (slow CVs, long latencies and low amplitudes) were always positive.

The reference material (n = 717) used for imputation and Z-score calculations was gathered between 2012 and 2022 from healthy subjects, and from patients referred to one of two Departments of Neurology in Mid-Norway (Ålesund or Trondheim) for non-specific symptoms without known disease (malignancy, diabetes, connective tissue disease, etc.), and found to be free of any neurological diagnosis after examination. Identical methods and protocols were used in both laboratories. Regression models were fitted to the data. The mean age was 45 years (SD 15 years, range 13-86 years), mean height was 171 cm (SD 7 cm, range 149-198 cm) and 70% were female. With regard to the sural nerve, differences in the NCS protocol (antidromic *vs* orthodromic measurements) did not allow for the reference material to be applied to patients from Oslo and Stavanger. For these patients, reference values for sural nerve conduction and sensory amplitudes were created by use of the Me-Ref method[2]. The Me-Ref method was applied to historical records from patients referred to the two hospitals' clinical neurophysiology laboratories over the last ~20 years (*n valid measurements* ≈ 40,000). Regression models were fitted for each NCS measure, adjusted for age and height. To ensure good fit in the upper and lower ends of the model, separate regression models were made for ages 18-49 and 50-70.

The final compound Z-score included the tibial and peroneal motor nerves (distal amplitude, conduction velocity, F-M min latency), the sural nerve (amplitude and conduction velocity), the peroneal superficial nerve (amplitude and conduction velocity), and the medial tibial plantar nerve (amplitude).

1 Van den Bergh PYK, van Doorn PA, Hadden RDM, et al. European Academy of Neurology/Peripheral Nerve Society guideline on diagnosis and treatment of chronic inflammatory demyelinating polyradiculoneuropathy: Report of a joint Task Force—Second revision. Journal of the Peripheral Nervous System 2021;26:242-268.

2 Nandedkar SD, Stålberg EV, Barkhaus PE. MeRef: Multivariable extrapolated reference values in motor merve conduction studies. Muscle & Nerve 2021;63(5):737-44. doi: https://doi.org/10.1002/mus.27195
